# The Impact of Host Genotype, Intestinal Sites and Probiotics Supplementation on the Gut Microbiota Composition and Diversity in Sheep

**DOI:** 10.3390/biology10080769

**Published:** 2021-08-12

**Authors:** Xiaoqi Wang, Zhichao Zhang, Xiaoping Wang, Qi Bao, Rujing Wang, Ziyuan Duan

**Affiliations:** 1 Hefei Institutes of Physical Science, Chinese Academy of Sciences, Hefei 230031, China; xqwang@genetics.ac.cn; 2 Science Island Branch of Graduate School, University of Science and Technology of China, Hefei 230026, China; 3 Institute of Genetics and Developmental Biology, Chinese Academy of Sciences, Beijing 100101, China; zczhang@genetics.ac.cn (Z.Z.); nxuwxp321@163.com (X.W.); baoqi2014@126.com (Q.B.)

**Keywords:** gut microbiota, composition, intestinal sites, sheep breeds, *Bifidobacterium*, feces

## Abstract

**Simple Summary:**

Given the fact that sheep serve as an increased meat product consumption in China, as well as a suitable model for fat deposition, their digestive tract microbiota has drawn growing attention. Our study depicted the gut microbiota community composition and diversity of sheep with varied genotypes but shared geography, with different sampling intestinal sites and probiotics supplementation time. The results indicated the great difference of the gut microbiota in samples from different sheep breeds, various intestinal sites, and different probiotics feeding times. Additionally, all results implied the dominance of the host factor in shaping unique microbiota under a certain environment, the greatest similarity of colonic and fecal microbiota, and the oral probiotic effectiveness for a given period of time for sheep.

**Abstract:**

Three sampling strategies with a 16s rRNA high-throughput sequencing and gene expression assay (by RT-PCR) were designed, to better understand the host and probiotics effect on gut microbiota in sheep. Sampling: (1) colon contents and back-fat tissues from small-tailed Han sheep (SHS), big-tailed Hulun Buir sheep (BHBS), and short-tailed Steppe sheep (SHBS) (*n* = 12, 14, 12); (2) jejunum, cecum and colon contents, and feces from Tan sheep (TS, *n* = 6); (3) feces from TS at 4 time points (nonfeeding, 30 and 60 feeding days, and stop feeding 30 days) with probiotics supplementation (*n* = 7). The results indicated SHS had the highest *Firmicutes* abundance, the thinnest back-fat, and the lowest expression of *C/EBPβ*, *C/EBPδ*, *ATGL*, *CFD*, and *SREBP1*. Some bacteria orders and families could be potential biomarkers for sheep breeds with a distinct distribution of bacterial abundance, implying the host genotype is predominant in shaping unique microbiota under a shared environment. The microbiota diversity and *Bifidobacterial* populations significantly changed after 60 days of feeding but restored to its initial state, with mostly colonies, after 30 days ceased. The microbiota composition was greatly different between the small and large intestines, but somewhat different between the large intestine and feces; feces may be reliable for studying large intestinal microbiota in ruminants.

## 1. Introduction

There have already been many researches and achievements demonstrating the importance of gastrointestinal tract bacteria, which plays an important role in the growth, metabolism, and immunity of hosts [1,2]. Especially the intestinal microbiota composition and stability, containing a temporal and spatial distribution status of bacteria [3], have a close relation with the host’s health and production performance of economically important animals [4]. It is then required to understand the intestinal microbial composition and its perturbations among economically important species, due to the production of enzymes for the digestion of cellulose and other plant polysaccharides, as well as the synthesis of certain vitamins [4,5].

Recent studies reported that diet, environment, and host genetic factors were important in maintaining or affecting intestinal microbiota composition [6,7,8]. Nevertheless, with the object and region of study expanding, the extent of influence on the microbiota composition and diversity remains controversial. A research, based on 709 cattle, indicated the effect of host genetics on rumen microbiota [9], but less research focused on sheep. In this study, we employed four types of sheep breeds, which are the small-tailed Han sheep (SHS), the big-tailed Hulun Buir sheep (BHBS), the short-tailed Steppe sheep (also named the small-tailed Hulun Buir sheep, SHBS), and the Tan sheep (TS) to explore how the host genetic background and dietary supplement impact the intestinal microbiota composition and stability in sheep (Table S1). These four types of sheep breeds originated from different types of wild ancestors [10,11,12], but all belonged to the Mongolian sheep group, according to their development and breed formation. The genetic differentiation relationship of SHS, BHBS, SHBS, and TS, analyzed by microsatellite loci, also supports that the 4 sheep breeds originated from Mongolian sheep, which is consistent with the history of breed formation [13]. Owing to similar appearance and totally different tail shape, BHBS and SHBS are the great model to study fat deposition [10], while SHS is one of the most widely distributed sheep breeds in China, due to the strong reproductive capacity, hereditary stability, and better adaptation [11]. To this day, BHBS, SHBS, and TS are the main meat sheep in Hulun Buir region and Ningxia Hui Autonomous Region in China.

Previous studies have reported gastrointestinal microbial community and diversity differences along the gastrointestinal tract of sheep [14]. Although the differences between small and the large intestinal microbiota are parallel, the great controversy was whether feces could represent large intestinal contents on the intestinal microbiota study [15,16]. Here, with the expectation of clarifying this issue, we selected TS to compare the similarities and differences of intestinal microbiota composition among different intestinal sites.

According to the point of ecological view, diet could select microbial communities within the gut ecosystem [17], but the effect of dietary components on the stability of the gut microbiota has been poorly addressed [18]. Antibiotics, as a dietary supplemental component, would put on a prominent selective pressure on the gut microbiota and induce the most drastic perturbations as members of the gut microbiota [19]. Current studies on probiotics, modulating the intestinal microbiota via oral administration in monogastric animals [20] and rumen microbiota of ruminants [21,22], have been reported many times, but the effect and acting time of probiotics remain highly uncertain after by-passing the rumen of ruminants. It has been reported that *Bifidobacterium* supplementation can affect lipid metabolism or decrease pathogenic microbes [23], and thereby affects intestinal microstability, hence the correct acting time and effectiveness of *Bifidobacterium* would play a meaningful role in the sheep gut ecosystem and its productive performance.

With ongoing studies on gastrointestinal tract microbiota, the above are common issues in sheep production and research. Therefore, we suggested the hypothesis: (1) host genetics influence intestinal microbial features that could relate to host productive performance of sheep in the same habitat; (2) fecal samples could substitute for large intestinal contents, to a certain extent, when characterizing large intestinal microbiota; (3) there has been a specific time for the onset and duration of oral *Bifidobacterium* in sheep. In this study, we have attempted to verify the hypothesis by 3 sampling strategies and provided reliable reference for sheep production and research.

## 2. Materials and Methods

### 2.1. Animals and Sampling

1. Samples from 3 different sheep breeds, with each breed belonging to one group. Colon contents and back-fat samples were collected from SHS (*n* = 12), BHBS (*n* = 14), and SHBS (*n* = 12), which were raised concurrently in the same manage condition, with the same forage in the Breeding Sheep Farm of state-owned Bayan Farm (Inner Mongolia Autonomous Region, China) from 4-month to 8-month age. The final body weights (FBW) ± SD of SHS, BHBS, and SHBS were 40.38 ± 6.00 kg, 38.90 ± 5.37 kg, and 37.47 ± 5.77 kg, respectively.
2. Samples from different intestinal sites. TS (female, 4-month-old, *n* = 6) were purchased from Ningxia livestock farm (Ningxia Hui Autonomous Region, China) and fed with commercial diets and raised in the same condition. After feeding 3 months, the intestinal content samples were collected from jejunum, cecum, colon, and feces from TS with 33.80 ± 4.20 kg (FBW ± SD), (Figure S1a).
3. Samples from sheep with probiotics administration, along with feeding. TS (female, *n* = 7) were purchased from Ningxia livestock farm (Ningxia Hui Autonomous Region, China) and fed with freeze-dried *bifidobacteria* (10^10^ CFU/g, 0.03% of basal diets), which was mixed into commercial diets. Sampling feces at T1 (4-month-old, 24.80 ± 1.22 kg, nonfeeding *bifidobacteria*), T2 (5-month-old, 30.90 ± 1.42 kg, feeding *bifidobacteria* 30 days), T3 (6-month-old, 36.68 ± 1.99 kg, feeding *bifidobacteria* 60 days), and T4 (7-month-old, 40.11 ± 2.14 kg, after stop feeding *bifidobacteria* 30 days). Due to the unexpected death of a sheep, there were 6 samples of T4 group (Figure S1b).

Fecal samples were immediately caught by hand covered with a sterile glove when the sheep defecated. Other intestinal contents and back-fat tissues were collected immediately after slaughter. The above samples were gathered in sterile tubes, cryopreserved immediately in liquid nitrogen, and transported to the laboratory for preservation. Diet samples were sent to Ningxia Feed Engineering and Technology Research Center (Ningxia Hui Autonomous Region, China) to analyze the chemical compositions (Table S2).

### 2.2. DNA Isolation and 16S rRNA Sequencing

Microbial genomic DNA of all samples were isolated using the TIANamp stool DNA kit (Tiangen, Beijing, China), the V3-V4 regions of bacterial 16S rRNA were amplified (using the 341F/805R primer set) and sequenced on an Illumina HiSeq2500 platform (Health Genomics Bioinformatics Technology Co., Ltd., Beijing, China).

### 2.3. RNA Extraction and Reverse Transcription Polymerase Chain Reaction (RT-PCR)

Total back-fat tissue RNA was extracted via a Tissue RNA Purification Kit (EZBioscience, Roseville, CA, USA) and was reversely transcribed to cDNA using HiScript^®^ III RT SuperMix (Vazyme Biotech, Nanjing, China). The mRNA expression levels of *IRX3*, *THY1*, *PLIN4*, *LPL*, *CFD*, *SREBP1*, *C/EBPα*, *C/EBPβ*, *C/EBPδ*, and *ATGL* genes were checked with iTaq^TM^ Universal SYBR^®^ Green Supermix (Bio Rad, Hercules, CA, USA) on a real-time PCR (CFX Connect, Bio Rad, Hercules, CA, USA), and data were analyzed by the _ΔΔ_Ct method with β-actin as the internal reference. All primers and thermocycling conditions were followed as Table S3 [24,25,26,27]. Each qPCR reaction was repeated in triplicate.

### 2.4. Processing of 16S rRNA Sequencing Data

The QIIME1.9.1 [28] was performed for quality filtering and demultiplexing. Chimeric sequences were removed using USEARCH61, and the remaining tags were clustered into OTUs, using UCLUST [29] with a cutoff of 97% similarity, OTUs representative sequences were taxonomically classified using the Ribosomal Database Project (RDP) [30] within QIIME and the 2013 Greengenes (13_8 release) database [31]. Then, the alpha diversity (Shannon diversity, phylogenetic diversity, and Chao1 index) and the beta diversity (Bray Curtis matrix) were calculated by QIIME1.9.1. Linear discriminant analysis (LDA) effect size (LEfSe) was used to select the bacterial markers of microbiota among groups, with the significantly different represented by LDA score ≥2.0 [32]. Analysis of similarities (ANOSIM) of fecal microbiota, based on the Bray Curtis similarity matrix, were generated using the vegan package of the R4.0.3. While the functional potential of the gut microbiota community was analyzed using PICRUSt [33], based on the OTUs clustered from 16S rRNA sequencing data, the metabolic predictions were identified from Kyoto Encyclopedia of Genes and Genomes (KEGG) database. Scientific graphs were generated by the ggplot2 package of R4.0.3.

### 2.5. Statistical Analysis

The relative mRNA expression levels of fat deposition, related genes and the relative abundance of bacteria, were presented as the mean ± standard deviation (SD). Alpha diversity and the relative abundance of bacteria for statistics were analyzed with one-way ANOVA by the SPSS21 (IBM, Armonk, NY, USA); differences were considered as significant at *p* < 0.05 and highly significant at *p* < 0.01. Based on PICRUSt predicted output files, the functional profiles analysis was processed with Welch’s *t*-test, with FDR correction in STAMP2.1.3. The Benjamini-Hochberg FDR method was employed to control false discovery rate and the significance was accepted at *p* < 0.05 [34].

## 3. Results

### 3.1. Bacterial Community Variation of BHBS, SHBS, and SHS in the Same Habitat

#### 3.1.1. Diversity and Taxonomic Analysis

**Figure 1:**
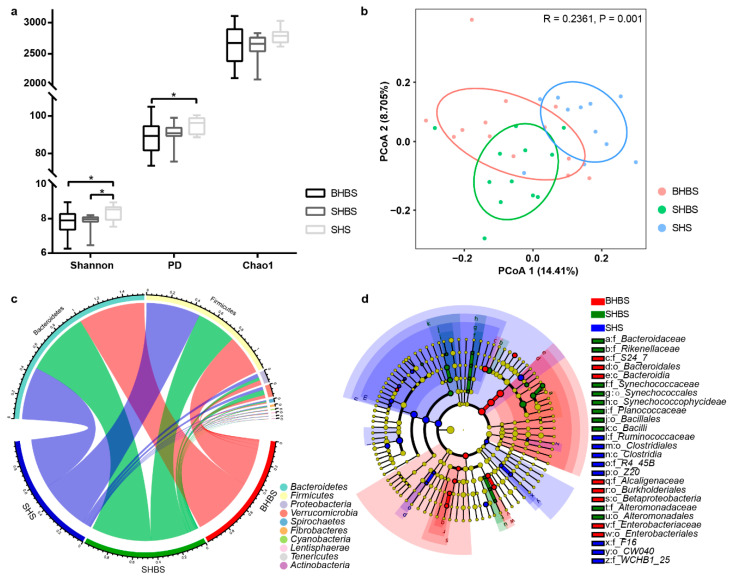

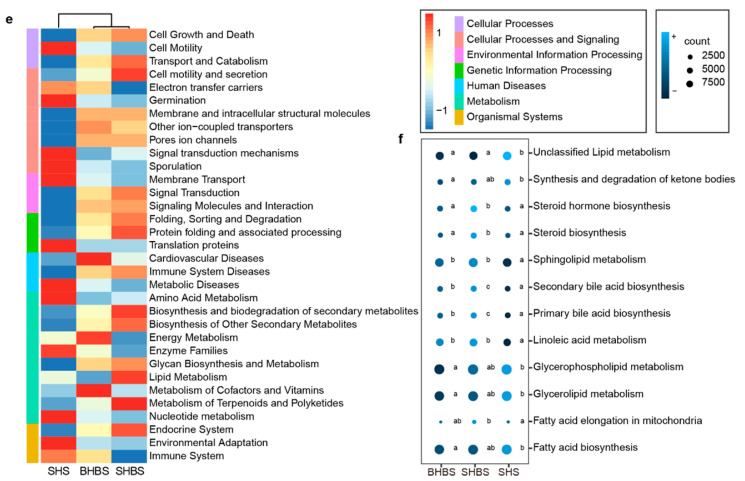
The different intestinal microbial communities between 3 sheep breeds. (**a**) Bacterial alpha diversity based on Shannon diversity, PD, and Chao1 index. * means the significant difference in statistics (*p* < 0.05). Shannon diversity represented microbial community diversity, Chao1 represented species richness, and PD (phylogenetic diversity) generated from species richness and evolutionary distance. (**b**) The Bray Curtis dissimilarity of colon microbiota from the 3 breeds were used for PCoA plot. Each dot represented the composition of the microbiota of each sample. Samples were grouped by colors as label showed. (**c**) The Circos plot displayed the bacterial taxonomic composition at phylum level. The abundance of phylum *Firmicutes* in SHS group was much higher than that in the other two breeds (*p* < 0.01), while the abundance of phylum *Bacteroidetes* in SHS group was much lower than that in the other two varieties (*p* < 0.01). (**d**) The cladogram of intestinal bacterial communities showed evolutionary relationship between 3 levels of taxonomy (class, order, and family) with LDA scores over 2, which demonstrated a significantly different abundance between breeds. (**e**,**f**) Showed the KEGG pathways (enriched the statistical discrepancies of OTUs number). Heat map generated from hierarchical clustering analysis of the normalized OTUs in Level_1&2 KEGG pathway. The scatter plot indicated the Level_3 pathway of lipid metabolism, lowercase was used to show statistical differences between 3 breeds, and bubble size represented the number of OTUs, while the deepen color stood for the diminishing tendency of enriched OTUs number among 3 groups.

In the study, we employed Shannon diversity, PD (phylogenetic diversity), and Chao1 index to represent the intestinal microbial flora alpha diversity (Figure 1a) to these sheep breeds. The results indicated that the higher the 3 diversity indexes, the greater the diversity of the communities. Based on the indexes of Shannon diversity, PD, and Chao1, the alpha diversity of intestinal microflora in SHS was the highest among 3 breeds; meanwhile, the evenness of SHS samples was much higher than SHBS and BHBS. Beta diversity of intestinal bacterial microbiota was performed by the principal coordinates analysis (PCoA), based on Bray-Curtis distance (Figure 1b). SHS and SHBS clustering separately indicated that the community structures of two breeds exhibited differences. As mentioned above, ANOSIM was employed to judge the difference of the microbiota community structure among breeds and indicated when pairwise comparison was carried out; there were significant differences between the BHBS and SHS groups and SHBS and SHS groups, and a larger variation existed within the BHBS group (Figure S2).

The line chart was created to show the correlation between OTU numbers and universal existence of OTU (Figure S3a). A Venn diagram (Figure S3b) showed a total of 393 OTUs (existed in ≥85% of each group’s population) were common among 3 groups, and 107, 86, and 95 unique OTUs were identified in the BHBS, SHBS, and SHS groups, respectively. Through comparing the composition of gut microbial communities (Figure 1c), the top 4 phyla were *Bacteroidetes*, *Firmicutes*, *Proteobacteria*, and *Verrucomicrobia*, existing among 3 breeds groups. Additionally, the relative abundance of phylum *Firmicutes* in the SHS group was much higher than that in the other two breeds, while the abundance of phylum *Bacteroidetes* in the SHS group was much lower than that in the other two varieties (*p* < 0.01) (Figure 1c and Figure S4). After performing LEfSe analysis, there were 12 families, 9 orders, and 5 classes with a LDA score over 2 (Figure 1d). Genera *CF231*, *Anaerofustis*, *Ruminobacter*, *Ruminococcus*, *Epulopiscium*, *Anaerostipes*, *SMB53*, and *Dorea* were differentially abundant in SHS, whereas the abundance of *Bacteroides*, *Lysinibacillus*, *Christensenella*, *Alteromonas*, *Prochlorococcus*, and *Butyricimonas* were enriched in SHBS. Moreover, *Blautia* and *Sutterella* in BHBS had LDA score over two (Figure S5).

#### 3.1.2. Functional Prediction and Lipid-Relevant Genes Detection

**Figure 2:**
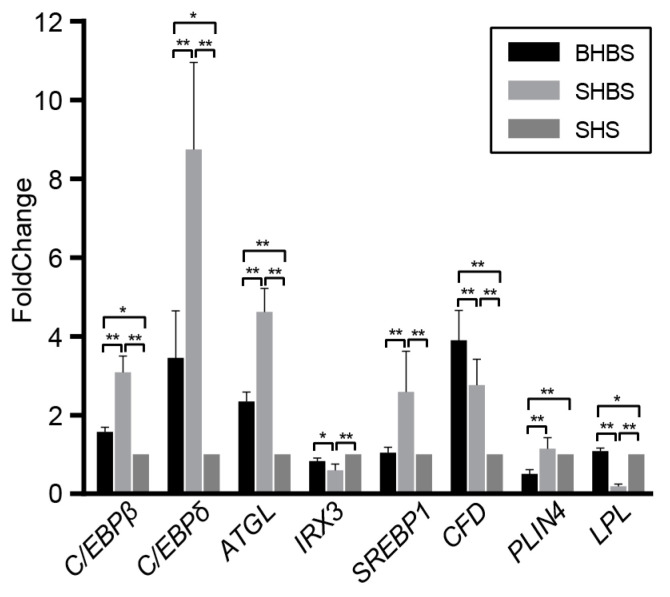
Relative mRNA expression levels of *C/EBPβ*, *C/EBPδ*, *ATGL*, *IRX3*, *SREBP1*, *CFD*, *PLIN4*, and *LPL* in 3 sheep breeds. The mRNA levels of genes involved in lipid metabolism were determined by qRT-PCR (analyzed by the _ΔΔ_Ct method with *β-actin* as the internal reference, ± SD). * means a significant difference at *p* < 0.05 level and ** means that at *p* < 0.01 level.

The enriched KEGG pathway of the gut microbial communities, in 3 breeds, were predicted by PICRUSt. As Figure 1e,f showed, the lipid metabolism pathway analysis showed that the intestinal microbial communities of SHBS and BHBS were more concentrated in fatty acid synthesis, glycerolipid and glycerophospholipid metabolism, ketone body synthesis, and the degradation pathway; the intestinal bacteria of SHS were more concentrated in primary and secondary bile acid synthesis, steroids and sphingolipids biosynthesis, and linoleic acid metabolism. These results tended to be consistent with the appearance of back-fat thickness of 3 sheep carcasses. When detecting the relative mRNA expression levels of *IRX3*, *THY1*, *PLIN4*, *LPL*, *CFD*, *SREBP1*, *C/EBPα*, *C/EBPβ*, *C/EBPδ*, and *ATGL*, which related to lipid metabolism, the expression of *C/EBPβ*, *C/EBPδ*, *ATGL*, and *CFD* were more highly expressed in the BHBS and SHBS groups than in the SHS group. In addition, a higher expression of *SREBP1* and a lower expression of *LPL* were found in the SHBS group than in other 2 breeds (Figure 2 and Figure S6).

### 3.2. Bacterial Community Variation in Different Intestinal Sites

**Figure 3:**
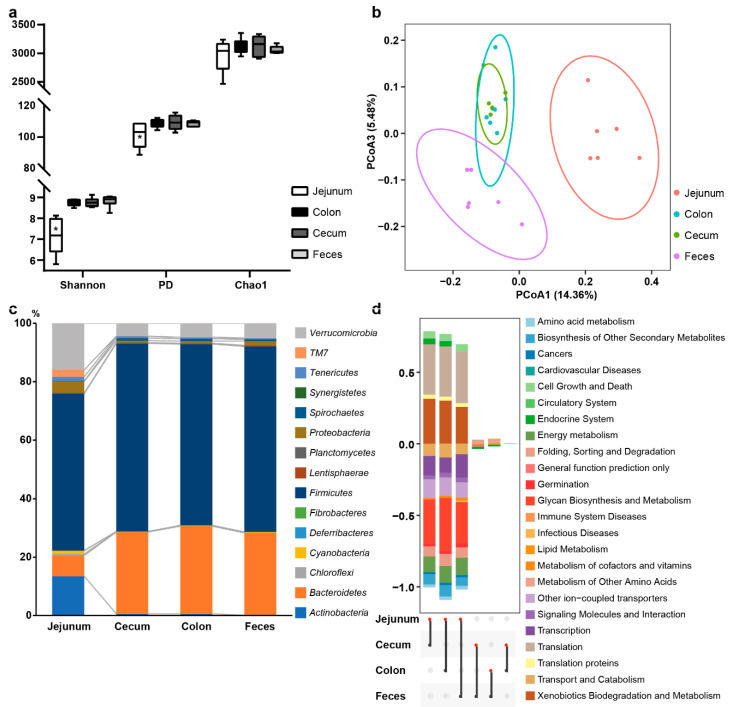
The similar and different of intestinal microbial communities in different intestinal sites. (**a**) Bacterial alpha diversity based on Shannon diversity, PD (Phylogenetic diversity), and Chao1 index. * on jejunum box indicates there was significant difference in alpha diversity between jejunum and other 3 groups (*p* < 0.05). (**b**) PCoA plot, based on the Bray Curtis dissimilarity of microbiota. Each dot represented the composition of the microbiota of each sample. Samples were grouped by colors as label showed. (**c**) Bar plot indicated the bacterial taxonomic composition and the change of relative abundance at phylum level of jejunum, cecum, colon, and feces. The microbial relative abundance of jejunum was significantly different from that of the other three sites at phylum level. (**d**) Stacked bar chart represented pathways (*n* = 24) with significant differences between 2 groups (bottom-colored spots). The upper x axis KEGG pathways displayed pathways, with greater number of OTUs in the red dot group than that in the black dot group, while that in the lower x axis was opposite. The height of each colored bar indicated the difference between the two groups.

The alpha diversity of microbiota among jejunum, cecum, colon, and feces, based on Shannon diversity, PD (phylogenetic diversity), and Chao1 index, were presented in Figure 3a. The jejunum had a significantly lower value at alpha diversity and the species evenness than the cecum, colon, and feces (*p* < 0.01), with similar alpha diversity index to each site. All samples displayed distinct clustering among small intestine (jejunum), large intestine (cecum, colon), and feces in PCoA, plotted with Bray-Curtis distance (Figure 3b), while cecum clustered into colon samples.

As shown in Figure 3c, the relative abundance of intestinal bacteria was calculated at the phylum level. *Bacteroidetes* and *Firmicutes* were the two predominant phyla in cecum, colon, and feces, while *Firmicutes*, *Verrucomicrobia*, *Actinobacteria*, and *Bacteroidetes* were the predominant phyla in jejunum. Nearly all bacterial phyla abundance in jejunum was significantly different from that in other 3 sites. Compared to cecum, phyla *Actinobacteri*, *Chloroflexi*, *Cyanobacteri*, *Proteobacteria*, *Tenericutes*, *TM7*, and *Verrucomicrobia* abundance in jejunum were significantly higher in colon and feces, moreover, the abundance of phyla *Bacteroidetes*, *Firmicutes*, and *Synergistetes* were lower (Figure S7). The genera abundance of jejunum was still difference from that of the other 3 sites, whereas the genera abundance sort in cecum, colon, and feces were same to each other. The top 10 genera were Unclassified_f_*Ruminococcaceae*, Unclassified_o_*Bacteroidales*, Unclassified_o_*Clostridiales*, Unclassified_f_*Lachnospiraceae*, Unclassified_f_*Bifidobacteriaceae*, *Ruminococcus*, Unclassified_f_*RFP12*, *5-7N15*, *Akkermansia*, and *Clostridium* in cecum, colon, and feces (Figure S8).

As the chart showed (Figure 3d), there was no significant difference in the gut microbial function between colon and cecum, while seven pathways (Level_2 KEGG pathway, endocrine system, immune system diseases, circulatory system, lipid metabolism, cardiovascular diseases, general function prediction only, and the metabolism of other amino acids) showed significant difference between the large intestine and feces. Compared with the other 3 sites, the bacteria of jejunum involved in “cellular processes and signaling” and “metabolism” were less.

### 3.3. Gut Bacterial Community Variation along with Feeding Time of Probiotics

#### 3.3.1. Analysis of the Fluctuation in Diversity and Bacterial Taxa

**Figure 4:**
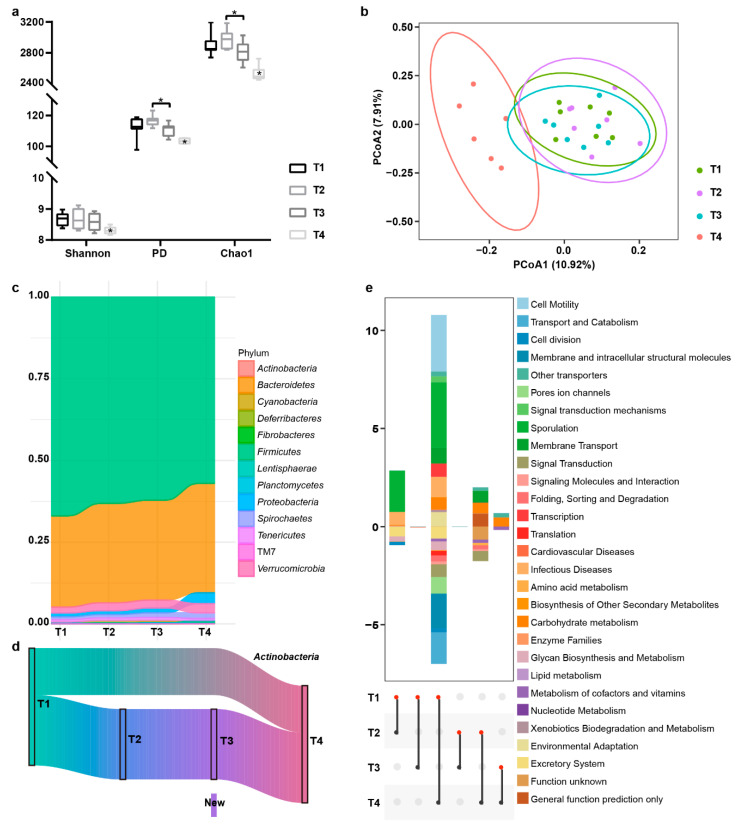
The correlation between intestinal microbial communities and probiotics feeding time. (**a**) Bacterial alpha diversity based on Shannon diversity, PD (Phylogenetic diversity), and Chao1 index. * on T4 box indicates the significant difference of T4 compared with other 3 groups (*p* < 0.05). (**b**) PCoA plot for the Bray Curtis dissimilarity of fecal microbiota from the 4 groups. Each dot represented the composition of the microbiota of each sample. Samples were grouped by colors as label showed. (**c**) Stream graph indicated the bacterial taxonomic composition and the change of relative abundance at phylum level from T1 to T4. Phyla *Firmicutes* and *Bacteroidetes* dominated the core microbiome in Tan breed sheep, phyla *Verrucomicrobia* and *Proteobacteria* followed. (**d**) OTUs that were shared in at least 50% of the population, at each sampling time, using Sankey plots, in *Actinobacteria*, which contained *bifidobacteria*. The heights of the black rectangles showed the relative number of OTUs at each sampling time, and the separated rectangle showed the emerging OTUs at T3 simply. (**e**) Stacked bar chart represented pathways (*n* = 29) with significant differences between 2 groups (bottom-colored spots). The upper x axis KEGG pathways displayed pathways with a greater number of OTUs in the red dot group than that in the black dot group, while that in the lower x axis was opposite. The height of each colored bar indicated the difference between 2 groups.

The T1, T2, T3, Shannon diversity, Chao1 index, and PD of Tan breed sheep were significantly lower at T4, in intestinal bacterial communities, while Chao1 and PD of bacterial communities at the T3 time point were significantly lower than those at the T2 time point (Figure 4a), indicating the higher species evenness of T3 than T2. Similarly, beta diversity of the intestinal bacterial community showed the distinction between the T4 samples and other 3 groups (Figure 4b). We examined the bacterial abundance, during timing points at the phylum level (Figure 4c), a total of 13 phyla, including *Cyanobacteria*, *Deferribacteres*, *Fibrobacteres*, *Firmicutes*, *Bacteroidetes*, *Proteobacteria*, *Lentisphaerae*, *Planctomycetes*, *Spirochaetes*, *TM7*, *Tenericutes*, *Verrucomicrobia*, and *Actinobacteria* were observed, with *Firmicutes* being the most abundant phylum, followed by *Bacteroidetes*, across each stage. With the prolongation of the feeding time with probiotics, the abundance of *Firmicutes* decreased, while *Bacteroidetes* increased, and both had significant differences between T1 and T4 (Figure S9). The probiotics (*Bifidobacterium*) belonged to phylum *Actinobacteria*, whereupon we were tracking individual OTUs within the *Actinobacteria*, to explore the temporal dynamics when time went by and found that the *Actinobacteria* fluctuated over time through. A small percentage of OTUs disappeared during probiotics administration, but reappeared after ending feed. After feeding probiotics for more than 30 days, probiotics (new OTU showed in Figure 4d) could be detected in feces. Interestingly, the trend of *Tenericutes* abundance was consistent with that of *Actinobacteria*. To identify which taxa were specific in feces on T1, T2, T3, and T4, respectively, and those core taxa in both time points, the UpSet and Venn analysis was performed on the OTUs that existed for at least 90% of the population at each group (Figure S10). Out of over 90% samples, 388 core species were unaffected by feeding and nonfeeding probiotics, while 72, 119, 105, and 103 specific OTUs appeared at 4 sampling time points, respectively. Time-associated bacterial taxa were identified by LEfSe, which displayed the significantly different abundance of bacteria (phylum to genus) among the 4 groups (Figure S11).

#### 3.3.2. Predictive Function Analysis

We also wondered which biological functions were affected by feeding or nonfeeding probiotics, through changing intestinal microbiota. Hence, the KEGG pathway comparison was performed to search for potential differences in the functions of the microbiota composition of feeding or nonfeeding probiotics. Figure 4e showed all significant KEGG pathways among 4 groups (*p* < 0.05), and there were many more differences between nonfeeding probiotics group (T1) and the group that stopped feeding probiotics after 30 days (T4) than in other groups. The microbiota of the T1 samples showed a higher abundance in the pathways of cell motility, sporulation, signal transduction mechanisms, other transporters, membrane transport, transcription, infectious diseases, carbohydrate metabolism, xenobiotics biodegradation and metabolism, lipid metabolism, and environmental adaptation than in the T4 samples; additionally, the microbiota of the T1 samples showed lower abundance in transport and catabolism, cell division, pores ion channels, membrane and intracellular structural molecules, signaling molecules and interaction, signal transduction, translation, folding, sorting and degradation, biosynthesis of other secondary metabolites, metabolism of cofactors and vitamins, glycan biosynthesis and metabolism, and excretory system than in the T4 samples. In addition, carbohydrate metabolism, other transporters, and nucleotide metabolism were significantly less abundant in T4 than T3; besides, the metabolism of cofactors and vitamins were significantly less abundant in T3 than T4. Likewise, sporulation, infectious diseases, enzyme families, carbohydrate metabolism, and lipid metabolism were significantly more abundant in T1 than T2, while amino acid metabolism, cell division, glycan biosynthesis and metabolism, and the excretory system were significantly more abundant in T2 than T1.

## 4. Discussion

### 4.1. Host Genotype Affected Intestinal Microbial Compositions in Sheep with Shared Geography

Generally, healthy individuals maintain a relatively stable structure of the gut microbiota, and we found that the phyla of *Bacteroidetes*, *Firmicutes*, *Proteobacteria*, and *Verrucomicrobia* were the core microbial in sheep. A recent study on Chinese populations revealed that geographical location and ethnicity are major two factors influencing the composition of the gut microbiota and geographical location has the greater influence [35], but how the ethnic difference existed is still unclear. A study on Merino sheep speculated that host genetics likely plays some role in developing unique gut microbiota [36]. A relevance study on cattle SNPs and rumen bacterial community composition also demonstrated that host genetics affected the rumen microbiota [37]. According to the Venn diagram in our study, although there were large number of common OTUs, the unique OTUs still existed among different breed groups in one habitat, implying that genotype would be the major factor affecting the gut microbiota in hosts living in same geographical location, with consistent nutrient diet.

Besides, the alpha diversity provided additional verification to the results of the association between host genetic background and bacterial community variation. As shown in Figure 1, the lowest dispersion degree of alpha diversity indexes in SHS revealed that the intestinal microbial was steadier, compared with that in SHBS and BHBS. This might contribute to the fact that SHS had been breeding relatively longer and more restrictively in the area of origination (from the 1980s) than two others and have a more accordant genotype or genetic background [11]. The pair-wise genetic differentiations, based on the D-loop sequence of mtDNA and microsatellite markers, showed a certain extent of genetic differentiation between SHS, SHBS, and BHBS [13,38]. Another possibility was that the breeding environment of SHS was warmer and more humid than Inner Mongolia, where BHBS and SHBS were raised, which would reduce the gut microbiota diversity [39]. It was unexpected that the distance of BHBS samples with the two other breeds were so close (PCoA plot), which could be inferred from the similar appearance (except tails shape) between BHBS and SHBS [10,40]; in addition, SHS, as one of the most widely distributed sheep breeds in China, might be introduced to BHBS group, in the artificial selection and breeding history.

Lots of studies revealed that *Firmicutes* and *Bacteroidetes* were the most predominant phyla in mammal gut and shared relative abundance with different species and breeds. There is a certain correlation between phylum *Firmicutes* and *Bacteroidetes* and fat deposition, both can promote fat deposition [41,42]. Though many reports indicated that obesity was related to an increased value of ratio for *Firmicutes* to *Bacteroidetes* in humans, rats, and pigs [8,41,43], there was still a report about a lean person with higher a value of ratio [42]. In our study, the value of ratio for phylum *Firmicutes* to *Bacteroidetes* in SHS was much higher than that in BHBS and SHBS (*p* < 0.01), which agrees with the report of a lean person above. According to multiple related reports about the ratio of *Firmicutes* to *Bacteroidetes* for obesity, it should be concluded that the fat deposition of SHS is greater than in Hulun Buir sheep, the actual observation and measurement from the carcass during the slaughter experiment in this study indicated that the 3 types of sheep breed backfat thickness (SHS = 2.44 mm, BHBS = 4.55 mm, and SHBS = 5.55 mm) were adverse to that correlation, under the same environment and feeding conditions, and showed the diminished proportion of *Firmicutes*, and an increase in *Bacteroidetes*, bring about a greater fat deposition in BHBS and SHBS, which is consistent with a recent study about SHS with a higher abundance of *Firmicutes* and lower abundance of *Bacteroidetes* than Tibetan, Dorset, and Dorper sheep [7]; the result was also confirmed by prediction analysis. PICRUSt metabolic predictions, based on the KEGG database, inferred that the fat metabolism of Hulun Buir sheep might have more inclination of the synthesis and deposition of body fat. The results of gene expression of fat metabolism were affirmed by the KEGG analysis too, which was in accordance with the carcass appearance of three types of sheep.

From our results, *Clostridiales* was the significant order-making SHS, having the highest abundance of *Firmicutes*, while *S24_7* was the significant family-letting BHBS, having the highest abundance of *Bacteroidetes*; families of *Bacteroidaceae*, *Rikenellaceae*, and *Synechococcaceae* were significant for SHBS, having the second highest abundance of *Bacteroidetes* among breeds. These bacterial families could be considered as anti-obesity markers; for example, *S24-7*, as an anti-obesity bacterium, could reduce the proportion of *Firmicutes* [44]. The lower abundance of *Bacteroidaceae* and *Rikenellaceae* were observed in obese people’s guts [45,46]. Most differentially abundant genera may be both indirectly and directly involved in lipid metabolism, such as *Christensenella*, *SMB53*, and *Blautia*, having a significant association with host genes *ALDH1L1*, *GNA12*, and *CD36*, which are involved in fatty acids metabolism [47], while *Dorea*, *Epulopiscium Ruminococcus*, and *Butyricimonas* were involved in fatty acids production [48,49]. Hence, fat deposition may be under the combined effect of gastrointestinal tract bacteria and their related host genes. However, it was a perplexity to explain why the breed with thickest backfat could house so many coexisting anti-obesity marker bacteria in SHBS and BHBS; rather, it might be the breed advantage of SHBS and BHBS, or it might be the fat deposition genes of the hosts, such as *IRX3*, *THY1*, *PLIN4*, *LPL*, *CFD*, *SREBP1*, *C/EBPα*, *C/EBPβ*, *C/EBPδ*, and *ATGL* [25,26] exerting a stronger effect on fat metabolism instead of intestinal microbiota.

### 4.2. The Relevance of Microbial Communities between Intestinal Sites

The results showed that the large intestine (cecum, colon) and feces had the significantly higher bacterial diversity and evenness, compared to small intestines (jejunum). The difference of microbial diversity between small and large intestines had been previously observed in Han sheep, cattle, elk, and swine, as well [14,50,51,52]. Much longer, higher concentrations of bile salt, digestive enzymes [14], and lower pH [53] might contribute to lower bacterial diversity in the small intestine. Besides that, the consensus in most studies was that *Firmicutes* and *Bacteroidetes* were the predominant phylum in the large intestine; this pattern was also observed, and is in agreement with, our results. Yet a bit different from the previous study of small intestines in sheep, in which they reported that *Firmicutes* and *Cyanobacteria* were dominant in jejunum of SHS [54], while higher relative abundances of *Firmicutes* and *Verrucomicrobia* were observed in jejunum of TS in our study. *Verrucomicrobia* had many functions such as improving glucose metabolism [55], it was one of the dominated phyla in feces of Tibetan [7] and Merino sheep [36]. The diversity distance of the bacterial community in cecum, colon, and feces was close to each other but still clustered into two groups; meanwhile, according to our study on the taxonomic distribution of bacterial abundance at phylum level, the bacterial abundance in feces was alike that in cecum and colon, except for phyla *Deferribacteres* and *Lentisphaerae*. The controversy about whether or not fecal samples can substitute the intestinal contents to explore intestinal microbiota resided in mammalian studies, and these research results were inherently ambivalent. Some studies supported that the feces can be representative of large intestine in bacterial study, whereas for healthy, antibiotics-receiving animals, the bacterial diversity in fecal and large intestinal samples were much alike [16], or the microbiota-related metabolite values were similar in feces and cecum [56]. While others held the view that microbiota of feces and large intestine sites were distinct, like intestinal disorders, the host effect could lead to differences in the bacterial community, between feces and large intestine [15,57], or even for variations of fecal bacterial taxa, which were not observed in horsy hindgut [58].

In our study, there were significant differences in bacterial composition from cecum, colon, and feces to jejunum. Generally, the highest microbial density and abundance tend to be in the large intestine, where was the usual site of microbial fermentation chamber for the substrates available to the microorganisms yet indigestible in the gastric region [53]. Our results showed that bacteria were more involved in the metabolism pathway of the large intestine than in that of the small intestine. Microbial degradation of the intractable digestive nutrients could maximize the absorption of nutrient by the large intestine of the host.

Integrating the predictive functional profiling of gut microbial communities, the study indicated that colon sampling is suitable for mammalian intestinal microbiota study. Fecal sampling could replace colon at the greatest similarity in distribution and abundance of microbial communities, but there are differences at 2 phyla level.

### 4.3. Dynamic Intestinal Microbial Communities Relevant to Probiotics Administration

With the daily probiotic treatment in diet administration, it produced little effect on its alpha and beta diversity during feeding time (T1–T3) for sheep intestinal bacteria. Despite this, the species richness was not changed over time; however, the species evenness was increased. When probiotics feeding stopped after 30 days (T4), the intestinal bacteria diversity decreased compared to administration periods. In the study, we found that probiotics could not colonize effectively until more than 30 days’ feeding. The intestinal bacterial community composition changed greatly with the duration of feeding. In the first 30 days (T2) of feeding probiotics, some number of intestinal bacteria changed, but the probiotics did not increase substantially. After feeding time lengthening over 30 days to 60 days (T3), the community of probiotics in intestine increased enormously, but discontinuation after 30 days for a cease administration (T4), and the probiotic decreased to no significant difference in intestine. Obviously, oral administration of probiotics can only colonize in the intestine temporarily, long-term continuous feeding could bring long-acting benefits.

In the study, we also observed the reduction of *clostridia* counts, led by administration of probiotics, which related to clostridial intestinal disorders [59] being clostridial pathogens transmit disease by forming spores [60]. This observation was in accordance with the results that probiotic administration can reduce clinical symptoms among person with symptomatic uncomplicated diverticular disease, meanwhile, maintain intestinal microflora balance in 60 days after the end of the therapeutic trial [61]. According to our predictive functional analysis, the pathway of sporulation, infectious diseases were significantly higher abundant in T4 than others indeed. Therefore, the reduction in community diversity may be caused by the decrease of harmful bacteria.

The other effect of probiotics administration was that the decreasing of abundance for *Firmicutes* and increasing for *Bacteroidetes* with the prolongation of feeding time. We inferred that impact of the ratio change would be continuous and could not disappear immediately with the cessation of probiotics feeding. The KEGG analysis showed the same impact as it did on the ratio changed. We also noticed that daily administration of probiotics were able to markedly reduce bacteria involved in carbohydrate metabolism and human disease, which were consistent with the reports on *Bifidobacteria* playing the key role in carbohydrate metabolism [62]. Hence, the result implied that probiotics like *Bifidobacterium* might be developed as dietary supplements against some diseases, such as psychiatric disease [63], asthma [64], and obesity [65]. Beyond the above, we still found that bacteria of *Tenericutes* (genus *Anaeroplasma*) and *Actinobacteria* (genus *Bifidobacterium*) had the coordinated variation with the ratio change of *Firmicutes* to *Bacteroidetes*. Then, more researches are needed to explore how long this influence will work and verify the effect on fat deposition.

## 5. Conclusions

Our results indicated that host genotype (breeds) played a major role in the maintenance of the intestinal bacterial community, especially in developing unique intestinal microbial compositions, and depicted the community composition and diversity of sheep intestinal microbiota conferring equilibrium among different host genotypes when in the same environment. From the perspective of animal production, the results facilitate studying the interaction between the host and gut microbial community and obtaining efficient gastrointestinal tract microbiota, related to animal performance.

Additionally, the bacterial community diversity of feces differed considerably from that in small intestine, whereas, it had resemblance to that in the large intestine; feces could be used to predict the large intestinal microbiota, relatively equal to its composition. Then, sampling feces, rather than large intestinal contents, is an effective and low-cost method for characterizing microbiota of large intestine.

When sheep’s gut microbiota was intervened, by the continuous supplement of probiotics, it would be perturbed with the administration duration and the structure of the communities would change towards a healthier state, although 60 days’ feeding is not sufficient as the threshold for a critical switch to an ecological stable transition, but is a transient one, and they restore the initial microflora. This non-permanent effect added a new view to probiotic supplementation and offered preliminary regularity for probiotics administration in sheep production.

## Data Availability

The datasets used and/or analyzed during the current study are available from the corresponding author on reasonable request.

## Supplementary Materials

The following are available online at https://www.mdpi.com/article/10.3390/biology10080769/s1, Table S1: information of 4 sheep breeds in this study, Table S2: main chemical composition of basal diets (DM basis), Table S3: the primer sequences for RT-qPCR, Figure S1: schematic diagrams of collecting samples from sheep, Figure S2: analysis of similarities (ANOSIM) of colon microbiota based on the Bray Curtis similarity matrix in 3 sheep breeds, Figure S3: Venn plot of the core OTUs in the fecal microbiota of 3 sheep breeds, Figure S4: bacterial phyla with significant differences in 3 sheep breeds, Figure S5: bacterial genera differences among 3 breeds using LEfSe, Figure S6: relative mRNA expression levels of THY1 and C/EBPα in 3 sheep breeds, Figure S7: bacterial phyla with significant differences in 4 sites of Tan breed sheep, Figure S8: the relative abundance of the bacterial taxa among jejunum, cecum, colon, and feces at genus level, Figure S9: sheep intestinal bacterial phyla in differences along with supplementing probiotics, Figure S10: the UpSet and Venn plots of core and specific OTUs in feces at 4 sampling times, Figure S11: the different abundance of bacteria (phylum to genus) among 4 timing points were identified by LEfSe.

## References

[1] Kataoka K. (2016). The intestinal microbiota and its role in human health and disease. J. Med. Investig..

[2] Bäckhed F., Ding H., Wang T., Hooper L.V., Koh G.Y., Nagy A., Semenkovich C.F., Gordon J.I. (2004). The gut microbiota as an environmental factor that regulates fat storage. Proc. Natl. Acad. Sci. USA.

[3] Costello E.K., Lauber C.L., Hamady M., Fierer N., Gordon J.I., Knight R. (2009). Bacterial Community Variation in Human Body Habitats Across Space and Time. Science.

[4] Clauss M., Hume I.D., Hummel J. (2010). Evolutionary adaptations of ruminants and their potential relevance for modern production systems. Animal.

[5] Hellstrand S. (2012). Animal production in a sustainable agriculture. Environ. Dev. Sustain..

[6] Sanchez-Morate E., Gimeno-Mallench L., Stromsnes K., Sanz-Ros J., Román-Domínguez A., Parejo-Pedrajas S., Inglés M., Olaso G., Gambini J., Mas-Bargues C. (2020). Relationship between Diet, Microbiota, and Healthy Aging. Biomedicines.

[7] Chang J., Yao X., Zuo C., Qi Y., Chen D., Ma W. (2020). The gut bacterial diversity of sheep associated with different breeds in Qinghai province. BMC Vet. Res..

[8] Bergamaschi M., Tiezzi F., Howard J., Huang Y.J., Gray K.A., Schillebeeckx C., McNulty N.P., Maltecca C. (2020). Gut microbiome composition differences among breeds impact feed efficiency in swine. Microbiome.

[9] Li F., Li C., Chen Y., Liu J., Zhang C., Irving B., Fitzsimmons C., Plastow G., Guan L.L. (2019). Host genetics influence the rumen microbiota and heritable rumen microbial features associate with feed efficiency in cattle. Microbiome.

[10] Zhi D., Da L., Liu M., Cheng C., Zhang Y., Wang X., Li X., Tian Z., Yang Y., He T. (2018). Whole Genome Sequencing of Hulunbuir Short-Tailed Sheep for Identifying Candidate Genes Related to the Short-Tail Phenotype. G3 Genes Genomes Genet..

[11] Cheng P. (1984). Livestock Breeds of China.

[12] Zhao Y. (2011). Sheep Production.

[13] Zhong T., Han J.L., Guo J., Zhao Q.J., Fu B.L., He X.H., Jeon J.T., Guan W.J., Ma Y.H. (2010). Genetic diversity of Chinese indigenous sheep breeds inferred from microsatellite markers. Small Rumin. Res..

[14] Wang J., Fan H., Han Y., Zhao J., Zhou Z. (2017). Characterization of the microbial communities along the gastrointestinal tract of sheep by 454 pyrosequencing analysis. Asian-Australas. J. Anim. Sci..

[15] Ingala M.R., Simmons N.B., Wultsch C., Krampis K., Speer K.A., Perkins S.L. (2018). Comparing Microbiome Sampling Methods in a Wild Mammal: Fecal and Intestinal Samples Record Different Signals of Host Ecology, Evolution. Front. Microbiol..

[16] Yan W., Sun C., Zheng J., Wen C., Ji C., Zhang D., Chen Y., Hou Z., Yang N. (2019). Efficacy of Fecal Sampling as a Gut Proxy in the Study of Chicken Gut Microbiota. Front. Microbiol..

[17] Byndloss M.X., Pernitzsch S.R., Bäumler A.J. (2018). Healthy hosts rule within: Ecological forces shaping the gut microbiota. Mucosal Immunol..

[18] Fassarella M., Blaak E.E., Penders J., Nauta A., Smidt H., Zoetendal E.G. (2021). Gut microbiome stability and resilience: Elucidating the response to perturbations in order to modulate gut health. Gut.

[19] Bhalodi A.A., van Engelen T.S.R., Virk H.S., Wiersinga W.J. (2019). Impact of antimicrobial therapy on the gut microbiome. J. Antimicrob. Chemother..

[20] Abd El-Hack M.E., El-Saadony M.T., Shafi M.E., Qattan S.Y.A., Batiha G.E., Khafaga A.F., Abdel-Moneim A.-M.E., Alagawany M. (2020). Probiotics in poultry feed: A comprehensive review. J. Anim. Physiol. Anim. Nutr..

[21] Hu Y., He Y., Gao S., Liao Z., Lai T., Zhou H., Chen Q., Li L., Gao H., Lu W. (2020). The effect of a diet based on rice straw co-fermented with probiotics and enzymes versus a fresh corn Stover-based diet on the rumen bacterial community and metabolites of beef cattle. Sci. Rep..

[22] Mani S., Aiyegoro O.A., Adeleke M.A. (2020). Characterization of Rumen Microbiota of Two Sheep Breeds Supplemented With Direct-Fed Lactic Acid Bacteria. Front. Vet. Sci..

[23] Gibson G.R., Roberfroid M.B. (1995). Dietary Modulation of the Human Colonic Microbiota-Introducing the Concept of Prebiotics. J. Nutr..

[24] Chi Y., Wang Y., Zhao Y., Zhu J., Lin Y. (2020). Study on Cloning and Expression Patterns of Goat C/EBPα, C/EBPβ and C/EBPδ Genes. Acta Agric. Boreali-Sin..

[25] Zhang Y., Li L., Wang Q., Zhan S., Wang L., Zhong T., Guo J., Zhang H. (2018). Fibroblast growth factor 21 induces lipolysis more efficiently than it suppresses lipogenesis in goat adipocytes. Cytotechnology.

[26] Fan H. (2016). Transcriptomic Difference Analysis for Tail Adipose Tissue of Hulun Buir Sheep. Ph.D. Dissertation.

[27] Liu L., Trent C.M., Fang X., Son N.H., Jiang H., Blaner W.S., Hu Y., Yin Y.X., Farese R.V., Homma S. (2014). Cardiomyocyte-specific Loss of Diacylglycerol Acyltransferase 1 (DGAT1) Reproduces the Abnormalities in Lipids Found in Severe Heart Failure. J. Biol. Chem..

[28] Caporaso J.G., Kuczynski J., Stombaugh J., Bittinger K., Bushman F.D., Costello E.K., Fierer N., Peña A.G., Goodrich J.K., Gordon J.I. (2010). QIIME allows analysis of high-throughput community sequencing data. Nat. Methods.

[29] Edgar R.C. (2010). Search and clustering orders of magnitude faster than BLAST. Bioinformatics.

[30] Cole J.R., Wang Q., Fish J.A., Chai B., McGarrell D.M., Sun Y., Brown C.T., Porras-Alfaro A., Kuske C.R., Tiedje J.M. (2014). Ribosomal Database Project: Data and tools for high throughput rRNA analysis. Nucleic Acids Res..

[31] DeSantis T.Z., Hugenholtz P., Larsen N., Rojas M., Brodie E.L., Keller K., Huber T., Dalevi D., Hu P., Andersen G.L. (2006). Greengenes, a Chimera-Checked 16S rRNA Gene Database and Workbench Compatible with ARB. Appl. Environ. Microbiol..

[32] Segata N., Izard J., Waldron L., Gevers D., Miropolsky L., Garrett W.S., Huttenhower C. (2011). Metagenomic biomarker discovery and explanation. Genome Biol..

[33] Langille M.G., Zaneveld J., Caporaso J.G., McDonald D., Knights D., Reyes J.A., Clemente J.C., Burkepile D.E., Vega Thurber R.L., Knight R. (2013). Predictive functional profiling of microbial communities using 16S rRNA marker gene sequences. Nat. Biotechnol..

[34] Parks D.H., Tyson G.W., Hugenholtz P., Beiko R.G. (2014). STAMP: Statistical analysis of taxonomic and functional profiles. Bioinformatics.

[35] Lin D., Wang R., Luo J., Ren F., Gu Z., Zhao Y., Zhao L. (2020). The Core and Distinction of the Gut Microbiota in Chinese Populations across Geography and Ethnicity. Microorganisms.

[36] Mamun M.A.A., Sandeman M., Rayment P., Brook-Carter P., Scholes E., Kasinadhuni N., Piedrafita D., Greenhill A.R. (2020). The composition and stability of the faecal microbiota of Merino sheep. J. Appl. Microbiol..

[37] Abbas W., Howard J.T., Paz H.A., Hales K.E., Wells J.E., Kuehn L.A., Erickson G.E., Spangler M.L., Fernando S.C. (2020). Influence of host genetics in shaping the rumen bacterial community in beef cattle. Sci. Rep..

[38] Sun W. (2017). Differential Analysis of Adipocyte Morphology and Fat Metabolism-Related Genes Expression in Two Strains of Hulun Buir Sheep. MA Dissertation.

[39] Bestion E., Jacob S., Zinger L., Di Gesu L., Richard M., White J., Cote J. (2017). Climate warming reduces gut microbiota diversity in a vertebrate ectotherm. Nat. Ecol. Evol..

[40] Fan H., Hou Y., Sahana G., Gao H., Zhu C., Du L., Zhao F., Wang L. (2019). A Transcriptomic Study of the Tail Fat Deposition in Two Types of Hulun Buir Sheep According to Tail Size and Sex. Animals.

[41] Turnbaugh P.J., Ley R.E., Mahowald M.A., Magrini V., Mardis E.R., Gordon J.I. (2006). An obesity-associated gut microbiome with increased capacity for energy harvest. Nature.

[42] Schwiertz A., Taras D., Schaefer K., Beijer S., Bos N.A., Donus C., Hardt P.D. (2010). Microbiota and SCFA in Lean and Overweight Healthy Subjects. Obesity.

[43] Zheng X., Huang F., Zhao A., Lei S., Zhang Y., Xie G., Chen T., Qu C., Rajani C., Dong B. (2017). Bile acid is a significant host factor shaping the gut microbiome of diet-induced obese mice. BMC Biol..

[44] Guo S., Zhao H., Ma Z., Zhang S., Li M., Zheng Z., Ren X., Ho C.-T., Bai N. (2020). Anti-Obesity and Gut Microbiota Modulation Effect of Secoiridoid-Enriched Extract from *Fraxinus mandshurica* Seeds on High-Fat Diet-Fed Mice. Molecules.

[45] Aguilar T., Nava G.M., Olvera-Ramírez A.M., Ronquillo D., Camacho M., Zavala G.A., Caamaño M.C., Acevedo-Whitehouse K., Rosado J.L., García O.P. (2020). Gut Bacterial Families Are Associated with Body Composition and Metabolic Risk Markers in School-Aged Children in Rural Mexico. Child. Obes..

[46] Li X., Li Z., He Y., Li P., Zhou H., Zeng N. (2020). Regional distribution of Christensenellaceae and its associations with metabolic syndrome based on a population-level analysis. PeerJ.

[47] Goodrich J.K., Davenport E.R., Beaumont M., Jackson M.A., Knight R., Ober C., Spector T.D., Bell J.T., Clark A.G., Ley R.E. (2016). Genetic Determinants of the Gut Microbiome in UK Twins. Cell Host Microbe.

[48] Pan Y.Y., Zeng F., Guo W.L., Li T.T., Jia R.B., Huang Z.R., Lv X.C., Zhang J., Liu B. (2018). Effect of Grifola frondosa 95% ethanol extract on lipid metabolism and gut microbiota composition in high-fat diet-fed rats. Food Funct..

[49] Huang S., Ji S., Yan H., Hao Y., Zhang J., Wang Y., Cao Z., Li S. (2020). The day-to-day stability of the ruminal and fecal microbiota in lactating dairy cows. Microbiologyopen.

[50] Mao S., Zhang M., Liu J., Zhu W. (2015). Characterising the bacterial microbiota across the gastrointestinal tracts of dairy cattle: Membership and potential function. Sci. Rep..

[51] Kim J.-H., Hong S.W., Park B.-Y., Yoo J.G., Oh M.-H. (2019). Characterisation of the bacterial community in the gastrointestinal tracts of elk (Cervus canadensis). Antonie Leeuwenhoek.

[52] Yang H., Huang X., Fang S., Xin W., Huang L., Chen C. (2016). Uncovering the composition of microbial community structure and metagenomics among three gut locations in pigs with distinct fatness. Sci. Rep..

[53] Karasov W.H., Douglas A.E. (2013). Comparative Digestive Physiology. Compr. Physiol..

[54] Zhang H., Shao M., Huang H., Wang S., Ma L., Wang H., Hu L., Wei K., Zhu R. (2018). The Dynamic Distribution of Small-Tail Han Sheep Microbiota across Different Intestinal Segments. Front. Microbiol..

[55] Shin N.R., Lee J.C., Lee H.Y., Kim M.S., Whon T.W., Lee M.S., Bae J.W. (2014). An increase in the *Akkermansia* spp. population induced by metformin treatment improves glucose homeostasis in diet-induced obese mice. Gut.

[56] Behr C., Sperber S., Jiang X., Strauss V., Kamp H., Walk T., Herold M., Beekmann K., Rietjens I.M.C.M., van Ravenzwaay B. (2018). Microbiome-related metabolite changes in gut tissue, cecum content and feces of rats treated with antibiotics. Toxicol. Appl. Pharmacol..

[57] Kozik A.J., Nakatsu C.H., Chun H., Jones-Hall Y.L. (2019). Comparison of the fecal, cecal, and mucus microbiome in male and female mice after TNBS-induced colitis. PLoS ONE.

[58] Grimm P., Combes S., Pascal G., Cauquil L., Julliand V. (2020). Dietary composition and yeast/microalgae combination supplementation modulate the microbial ecosystem in the caecum, colon and faeces of horses. Br. J. Nutr..

[59] Yang J., Yang H. (2020). Transcriptome Analysis of the Clostridioides difficile Response to Different Doses of Bifidobacterium breve. Front. Microbiol..

[60] Shen A., Edwards A.N., Sarker M.R., Paredes-Sabja D. (2019). Sporulation and Germination in Clostridial Pathogens. Microbiol. Spectr..

[61] Laghi L., Mastromarino P., Elisei W., Capobianco D., Zhu C.L., Picchio M., Giorgetti G., Brandimarte G., Tursi A. (2018). Impact of treatments on fecal microbiota and fecal metabolome in symptomatic uncomplicated diverticular disease of the colon: A pilot study. J. Biol. Regul. Homeost. Agents.

[62] Ali N., Gong H., Giwa A.S., Yuan Q., Wang K. (2019). Metagenomic analysis and characterization of acidogenic microbiome and effect of pH on organic acid production. Arch. Microbiol..

[63] Abildgaard A., Kern T., Pedersen O., Hansen T., Lund S., Wegener G. (2020). A diet-induced gut microbiota component and related plasma metabolites are associated with depressive-like behaviour in rats. Eur. Neuropsychopharmacol..

[64] Terada-Ikeda C., Kitabatake M., Hiraku A., Kato K., Yasui S., Imakita N., Ouji-Sageshima N., Iwabuchi N., Hamada K., Ito T. (2020). Maternal supplementation with Bifidobacterium breve M-16V prevents their offspring from allergic airway inflammation accelerated by the prenatal exposure to an air pollutant aerosol. PLoS ONE.

[65] Hossain M., Park D.S., Rahman M.S., Ki S.J., Lee Y.R., Imran K.M., Yoon D., Heo J., Lee T.J., Kim Y.S. (2020). Bifidobacterium longum DS0956 and Lactobacillus rhamnosus DS0508 culture-supernatant ameliorate obesity by inducing thermogenesis in obese-mice. Benef. Microbes.

