# Supplementary material for: The Impact of Host Genotype, Intestinal Sites and Probiotics Supplementation on the Gut Microbiota Composition and Diversity in Sheep
Source: Biology (Basel). 2021 Aug 12;10(8):769. doi: 10.3390/biology10080769 (PMC8389637; doi:10.3390/biology10080769)

Table S1 Information of 4 sheep breeds in this study.

Sheep breed/genotype	Origin and distribution	Reference
Short-tailed Steppe sheep (SHBS)	Rise from the post-Baikal, the former Soviet Union, and introduced into Inner Mongolia in 1918, and now is mainly distributed in Xini River Basin of Ewenki Autonomous Banner, Inner Mongolia.	[10]
Big-tailed Hulun Buir sheep (BHBS)	Indigenous breed of Inner Mongolia and mainly distributed in the grasslands of Xin Barag Zuoqi, Xin Barag Youqi and Chen Barag Qi, and its tail is significantly larger than that of the SHBS.	[10]
Small-tailed Han sheep (SHS)	The typical breed in China, which was introduced to the plateau in the 1980s and developed in the semi-humid agricultural areas (Henan, Hebei, Shandong, Anhui and Jiangsu Provinces) with wide adaptation.	[11]
Tan sheep (TS)	a Chinese indigenous sheep breed that Mongolian sheep migrated to Ningxia Provinces through nomadic migration and was bred on both sides of the Yellow River for generations.	[12]

Table S2 Main chemical composition of basal diets (DM basis).

Chemical composition, % of DM	Sampling 1	Sampling 2	Sampling 3
OM	91.31	90.49	89.89
CP	9.91	6.49	14.10
EE	1.92	1.75	1.27
ADF	29.67	28.36	28.40
NDF	53.72	44.90	38.82
GE, MJ/kg	17.21	15.76	16.43

OM, organic matter; CP, crude protein; EE, crude fat; ADF, acid detergent fiber; NDF, neutral detergent fiber; GE, Gross energy.

Sampling 1: bacterial community comparison in Colon contents among SHS group, BHBS group and SHBS group breeds.

Sampling 2: bacterial community comparison in host intestinal across different sampling sites.

Sampling 3: bacterial community variation in host intestinal along with feeding time of probiotics.

Table S3 The primer sequences for RT-qPCR.

Genes	Primers	Tm	References
β-actin	F:5’-AGGCCCAGAGCAAGAGAGGTA<br>R:5’-GGGGTGTTGAAGGTCTCAAACA		[25]
PLIN4	F:5’-CTTTCGAGCATGCCCTCA<br>R:5’-CTGCTCACATCCCTGACTGG		[24]
LPL	F:5’-TCCTACTTCAGCTGGTCCAA<br>R:5’-AGAGACTTGTCATGGCATTTCA		[24]
SREBP1	F:5’-CTGCTGACCGACATAGAAGACAT<br>R:5’-GTAGGGCGGGTCAAACAGG		[23]
C/EBPα	F:5’-CCGTGGACAAGAACAGCAA<br>R:5’-GGCGGTCATTGTCACTGG		[23]
C/EBPβ	F:5’-CAAGAAGACGGTGGACAAGC<br>R:5’-AACAAGTTCCGCAGGGTG	60℃	[22]
C/EBPδ	F:5’-GGAACCCGCTGCCTTCTAC<br>R:5’-AGGTCGGCGAAGAGCTCG		[22]
ATGL	F:5’-GGAGCTTATCCAGGCCAATG<br>R:5’-TGCGGGCAGATGTCACTCT		[23]
CFD	F:5’-TGCACCTGGTGGTTCTGATC<br>R:5’-CACCCACTGCTCTGCTATCA		[24]
IRX3	F:5’-GCTGTAGTGCCTTGGAAGTGGAG<br>R:5’-TAAGACCAGAGCGGCATCCAG		[24]
THY1	F:5’-AGGATGAGGGGACCTACACA<br>R:5’-TGAAGTCCATGGCCTGCA		[24]

Figure S1 Schematic diagrams of collecting samples from sheep. (a) There were 3 contents of intestinal sites (jejunum, cecum, colon) and feces collecting from sheep at almost the same time. (b) Diagram showed feces were sample at T1 (nonfeeding probiotics), T2 (feeding probiotics 30 days), T3 (feeding probiotics 60 days), T4 (stop feeding probiotics 30 days).

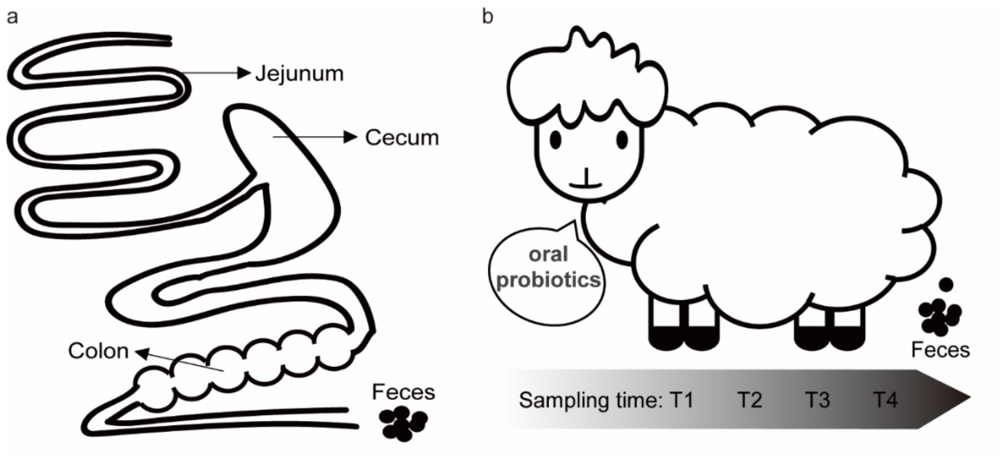

Figure S2 Analysis of similarities (ANOSIM) of colon microbiota based on the Bray Curtis similarity matrix in 3 sheep breeds. ANOSIM statistic R values indicate the differences between and within groups. P values show the significant different at $P < 0.05$.

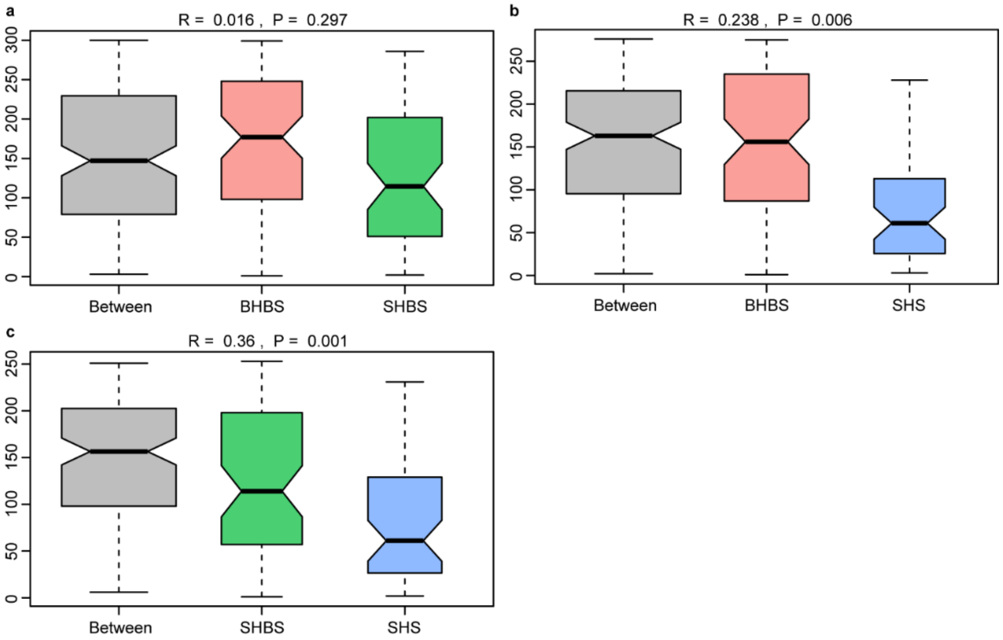

Figure S3 Venn plot of the core OTUs in the fecal microbiota of 3 sheep breeds. (a) The core OTU numbers can be observed in different fraction of samples. (b) The numbers in the Venn diagram indicated the number of OTUs which existed ≥ 85% of each group population.

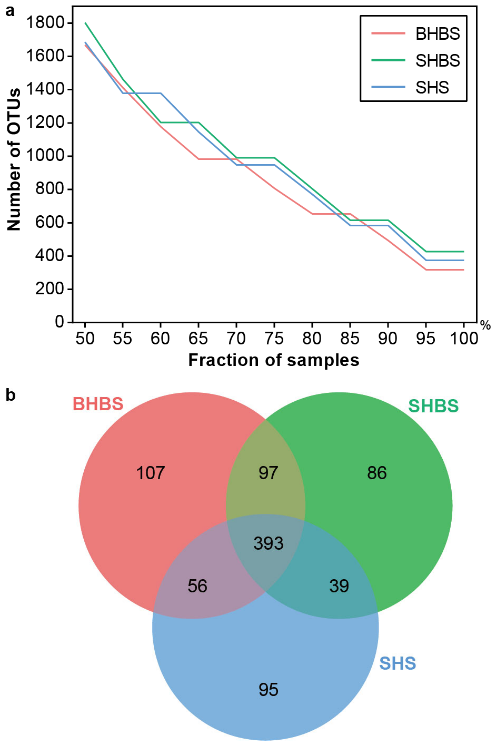

Figure S4 Bacterial phyla with significant differences in 3 sheep breeds. The asterisk means the significance at $P \leq 0.05$, and the 2 asterisks means significance level $P < 0.01$.

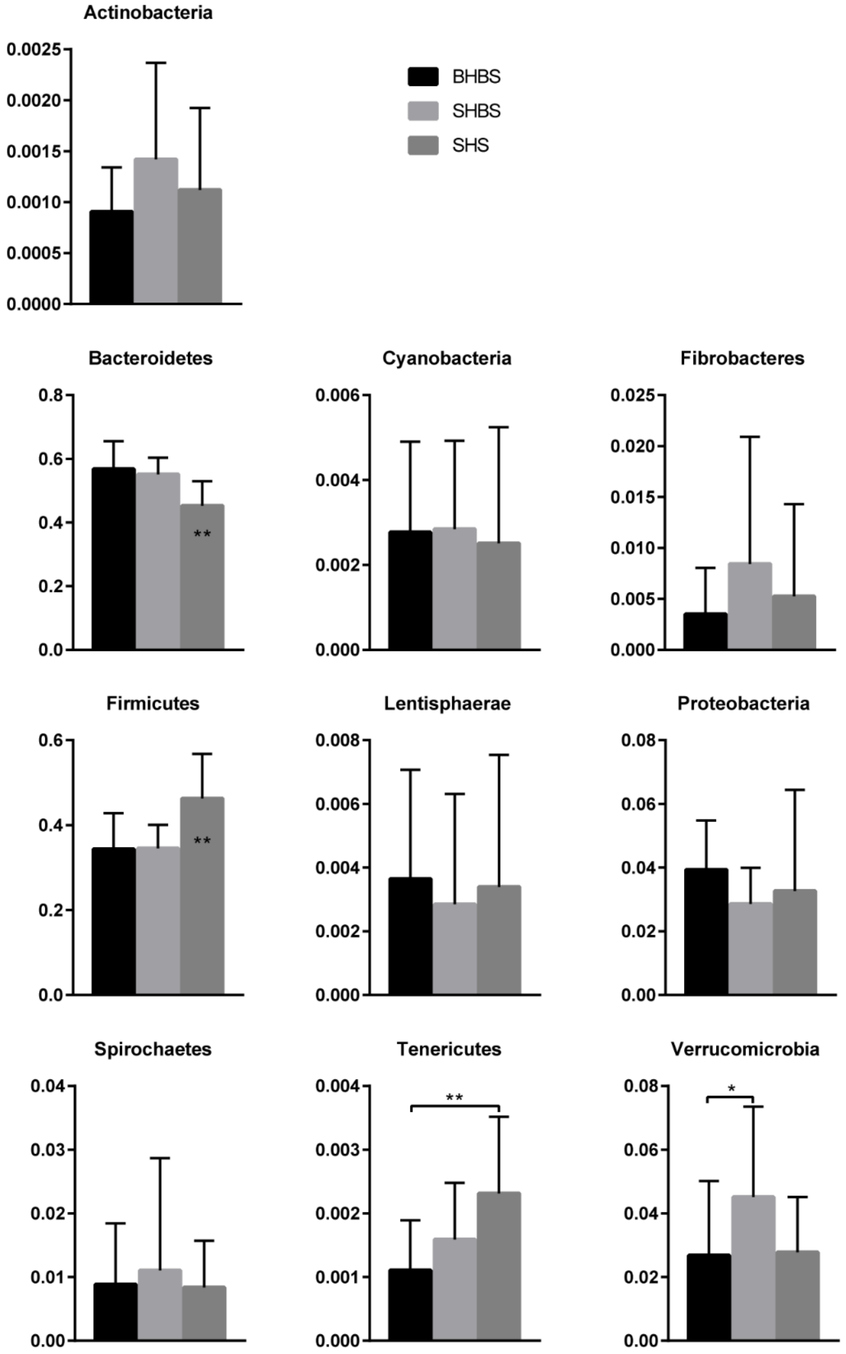

Figure S5 Bacterial genera differences among 3 breeds using LEfSe. It was significantly different when the logarithmic LDA score was over 2.0.

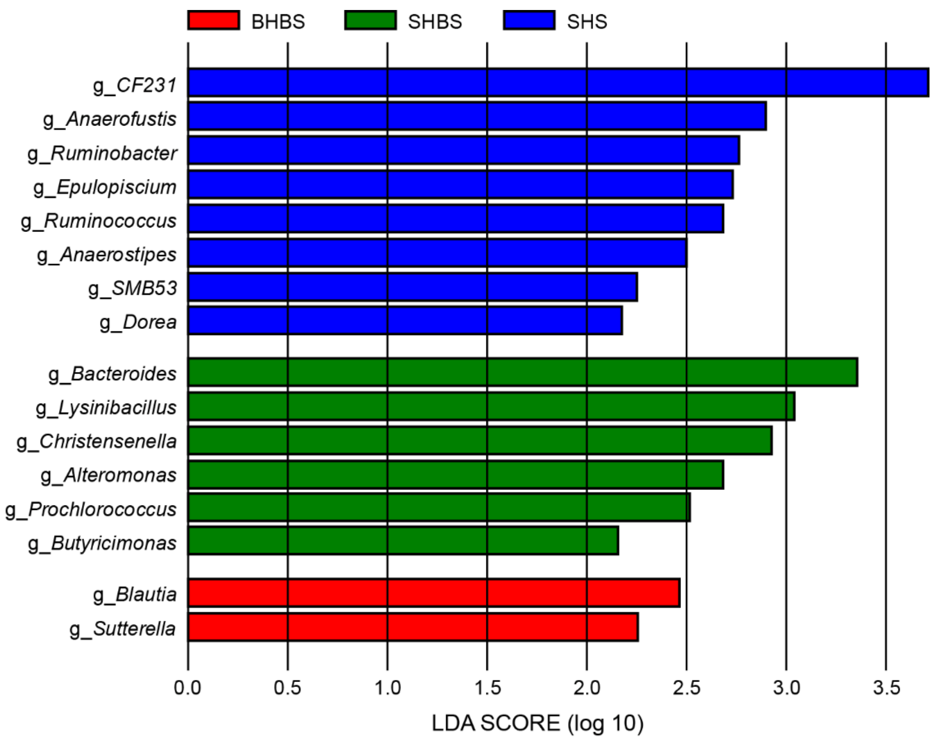

Figure S6 Relative mRNA expression levels of *THY1* and *C/EBPα* in 3 sheep breeds. The mRNA levels of genes involved in lipid metabolism were determined by qRT-PCR (analyzed by the ΔΔCt method with *β-actin* as the internal reference, ± SD).

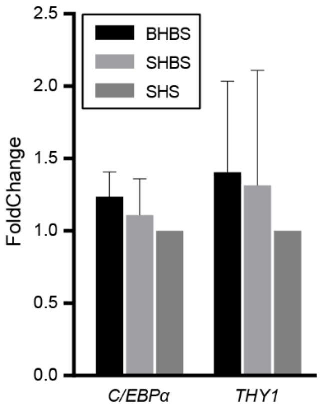

Figure S7 Bacterial phyla with significant differences in 4 sites of Tan sheep. The asterisk means the significance at $P < 0.05$, and the 2 asterisks means significance level $P < 0.01$.

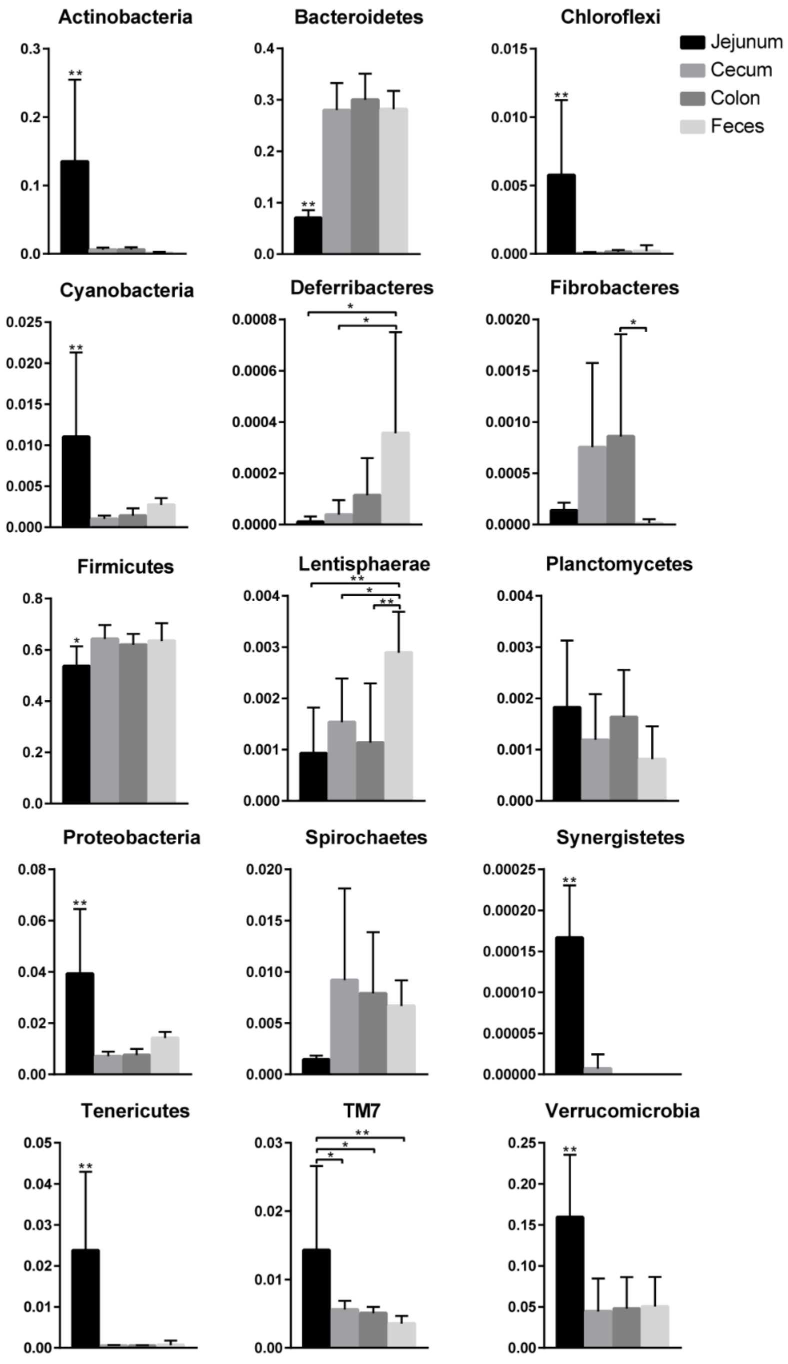

Figure S8 The relative abundance of the bacterial taxa among jejunum, cecum, colon, and feces at genus level. Bar plot indicated the bacterial taxonomic composition and the change of relative abundance at genus level of jejunum, cecum, colon, and feces.

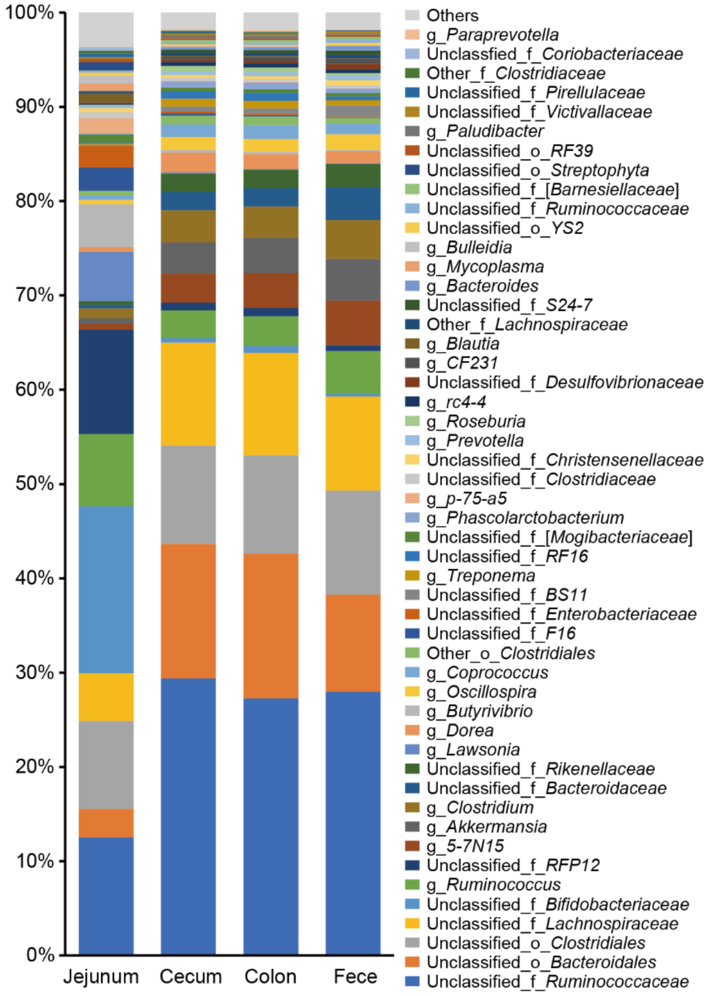

Figure S9 Sheep intestinal Bacterial phyla in differences along with supplementing probiotics. The asterisk means the significance at $P < 0.05$, and the 2 asterisks means significance level $P < 0.01$.

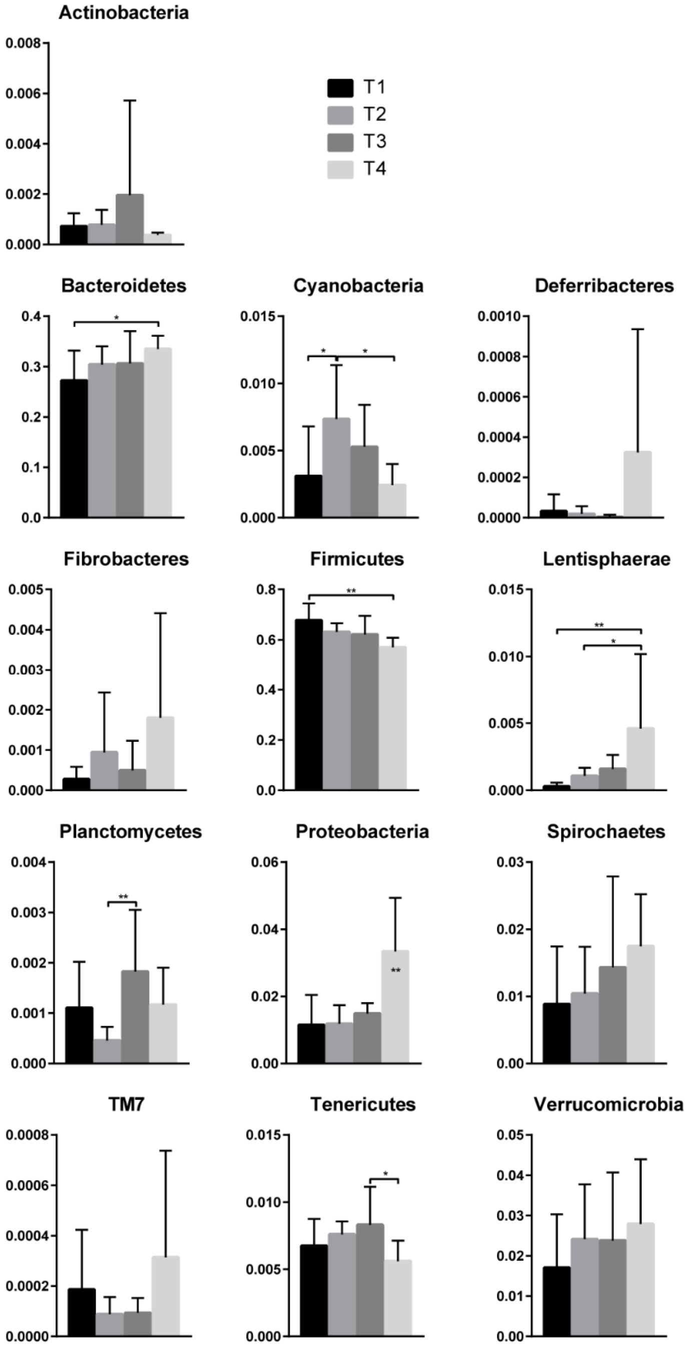

Figure S10 The Upset and Venn plot of core and specific OTUs in feces at 4 sampling time. T1 (nonfeeding probiotics), T2 (feeding probiotics 30 days), T3 (feeding probiotics 60 days), T4 (stop feeding probiotics 30 days). The numbers in the Upset and Venn plots indicated the number of OTUs which existed ≥ 90% of the population at each sampling time.

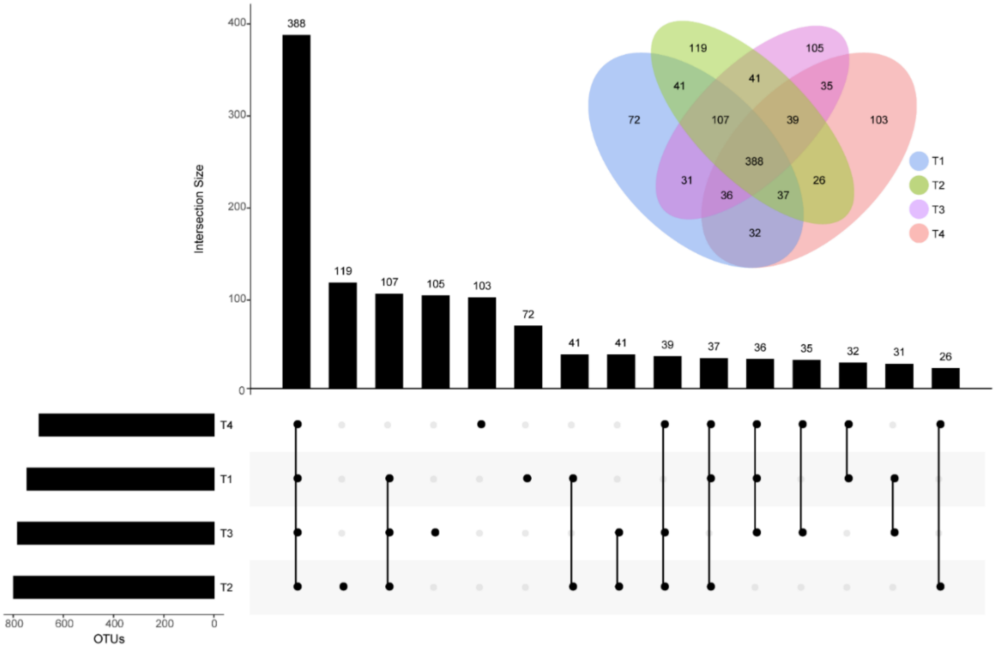

Figure S11 The different abundance of bacteria (phylum to genus) among 4 timing points were identified by LEfSe. It was significantly different when the logarithmic LDA score was over 2.0. The histogram showed LDA scores of differentially abundant taxa among 4 groups.

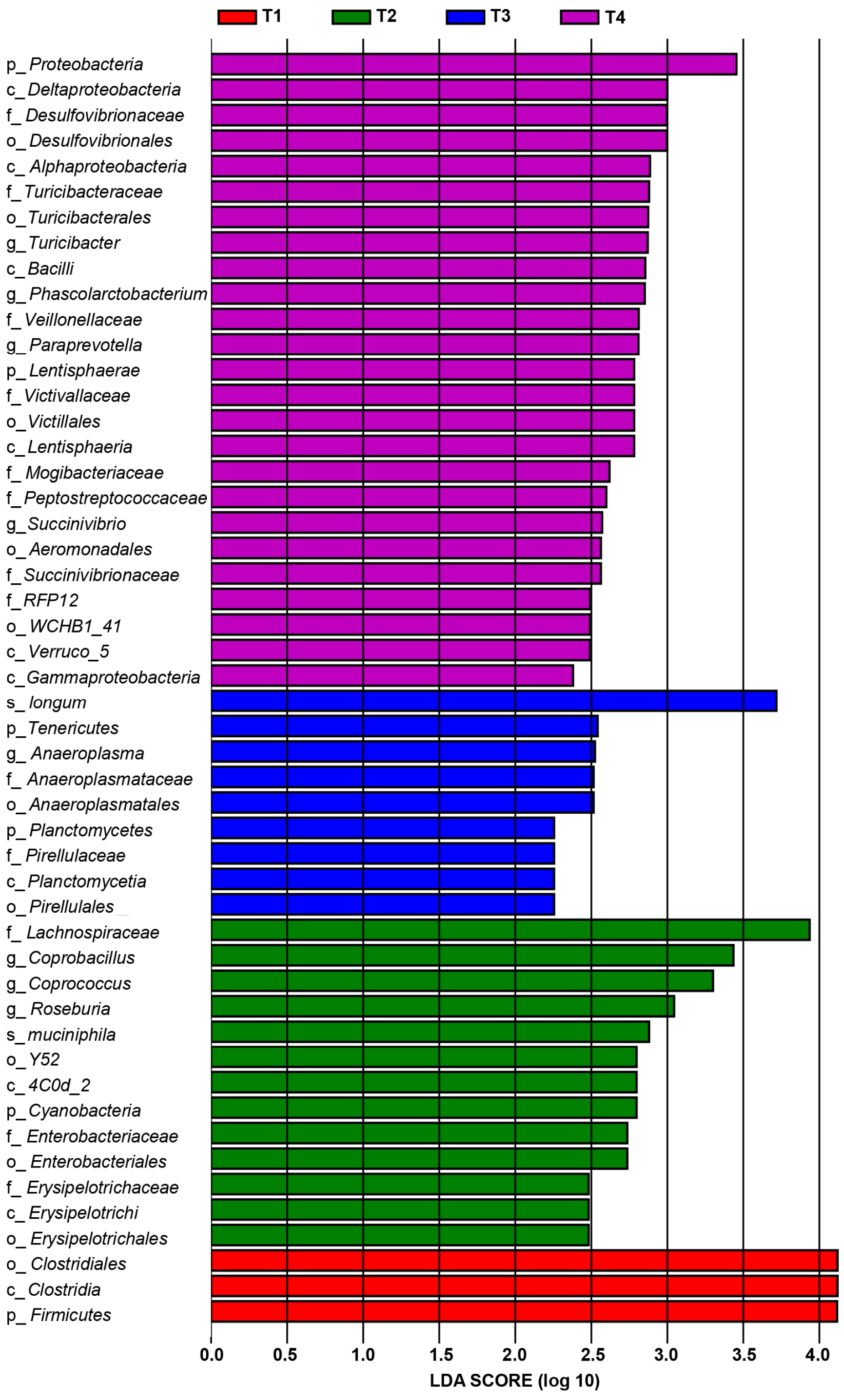

Supplement: Supplementary file 1 [file biology-10-00769-s001.zip › biology-1305164-supplementary.pdf]
